# Pancreatic Insulin Content Regulation by the Estrogen Receptor ERα

**DOI:** 10.1371/journal.pone.0002069

**Published:** 2008-04-30

**Authors:** Paloma Alonso-Magdalena, Ana B. Ropero, M. Pilar Carrera, Christopher R. Cederroth, Mathurin Baquié, Benoit R. Gauthier, Serge Nef, Enrico Stefani, Angel Nadal

**Affiliations:** 1 Instituto de Bioingeniería, Universidad Miguel Hernández de Elche, Alicante, Spain; 2 CIBER de Diabetes y Enfermedades Metabólicas Asociadas (CIBERDEM), Instituto de Salud Carlos III, Alicante, Spain; 3 Department of Genetic Medicine and Development, University of Geneva Medical School, Geneva, Switzerland; 4 Department of Cell Physiology and Metabolism, University of Geneva Medical School, Geneva, Switzerland; 5 Division of Molecular Medicine, Department of Anesthesiology, David Geffen School of Medicine at University of California Los Angeles, Los Angeles, California, United States of America; 6 Department of Physiology, David Geffen School of Medicine at University of California Los Angeles, Los Angeles, California, United States of America; University of Bremen, Germany

## Abstract

The function of pancreatic β-cells is the synthesis and release of insulin, the main hormone involved in blood glucose homeostasis. Estrogen receptors, ERα and ERβ, are important molecules involved in glucose metabolism, yet their role in pancreatic β-cell physiology is still greatly unknown. In this report we show that both ERα and ERβ are present in pancreatic β-cells. Long term exposure to physiological concentrations of 17β-estradiol (E2) increased β-cell insulin content, insulin gene expression and insulin release, yet pancreatic β-cell mass was unaltered. The up-regulation of pancreatic β-cell insulin content was imitated by environmentally relevant doses of the widespread endocrine disruptor Bisphenol-A (BPA). The use of ERα and ERβ agonists as well as ERαKO and ERβKO mice suggests that the estrogen receptor involved is ERα. The up-regulation of pancreatic insulin content by ERα activation involves ERK1/2. These data may be important to explain the actions of E2 and environmental estrogens in endocrine pancreatic function and blood glucose homeostasis.

## Introduction

Estrogen receptors are emerging as important molecules for glucose homeostasis [1]. It is known that ERα knock-out (ERαKO) mice are obese and insulin resistant [2]. ERα is implicated in β-cell survival [3]. It has been reported that estrogen function deficiency in men, due to the absence of ERα or aromatase, results in impaired glucose metabolism to such an extent that one patient developed type II diabetes mellitus [4]. Similar abnormalities were found in aromatase-deficient (ArKO) mice [5]. Recently, it has been described that both ERα and ERβ regulate the glucose transporter GLUT4 in skeletal muscle [6] and insulin sensitivity in the liver [7].

As described above, the role of estrogen receptors in glucose metabolism has been mainly studied in tissues other than the endocrine pancreas. However, the endocrine pancreas is crucial in the control of blood glucose homeostasis, because β-cells synthesize and release insulin, the only hormone able to reduce glycaemia. Therefore, to understand how estrogens affect β-cell adaptation to physiological situations such as pregnancy, it is essential to investigate the role of ERα and ERβ in this cellular system.

In addition to the role that estrogen receptors may play in the physiology of pancreatic β-cells, we should bear in mind that any disruption of β-cell physiology is associated with the development of type II diabetes mellitus. Indeed, recent epidemiological reports link the existence of endocrine disrupting chemicals (EDCs) in blood, such as persistant organic pollutants (POPs) [8] and phthalates [9], with type II diabetes and insulin resistance. In particular, the estrogenic pollutant Bisphenol-A (BPA) was described as altering pancreatic β-cell function and blood glucose homeostasis in mice [10].

BPA is the monomer employed to make polycarbonate plastic and resins used as lining for metal cans and as a plasticizer in other widely used plastics such as polyvinyl chloride (PVC) and polyethylene terephthalate (PET). Under normal conditions, this endocrine disruptor has been shown to leach from food and beverage containers [11], [12] as well as from dental sealants [13]. Widespread human exposure to significant amounts of BPA has been reported. For example, BPA was found in the urine of 95% of US citizens [14] and its concentration in human serum ranges from 0.2–1.6 ng/ml serum (0.88–7.0 nM) 15, 16.

The mechanism proposed for BPA actions has been based on its binding to ERα and ERβ, inducing estrogenic signals that modify gene expression [17]. However, the affinity of BPA for ERs was reported to be 10,000–100,000-fold lower than that of 17β-estradiol [18], [19] and it was shown to have a lower effect in several bioassays, such as the uterotrophic one [20]. Because of this, BPA has been considered to be a weak estrogen.

In this study, we demonstrate that doses of BPA within the range found in human serum, as well as physiological doses of E2 regulate pancreatic insulin synthesis in intact islets of Langerhans. This long-term action is mediated by the estrogen receptor ERα. These results shed light on the role of ERα in the physiology of the endocrine pancreas and its consequences on blood glucose homeostasis.

## Results

### Regulation of insulin content and secretion in intact islets of Langerhans by 17β-Estradiol

**Figure 1:**
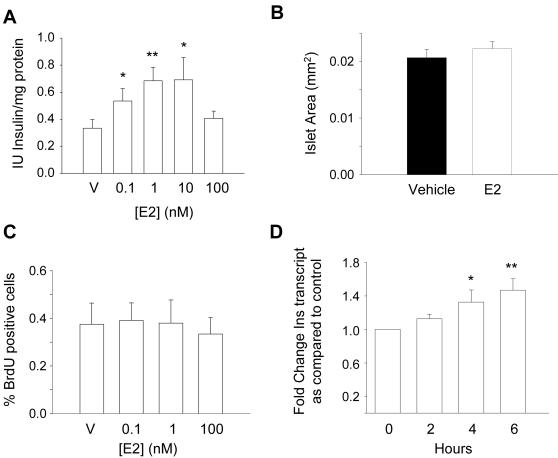
E2 action on insulin content, β-cell mass and insulin gene expression. (A) Insulin content of islets exposed to increasing doses of E2 for 48 hours, V means vehicle, (n = 8, 8 mice), * p<0.05, ** p<0.01, Mann-Whitney test, compared to vehicle. (B) Average size of islets cultured in the presence of vehicle (black column) or 1 nM E2 (white column) for 48 hours. Data represent the mean±SEM of at least 60 islets per condition obtained from 5 different animals. (C) Dispersed islet cells were counted under a fluorescent microscope and results are depicted as a percentage of BrdU-positive cells, values represent the mean±SEM of 4 independent experiments, each representing 3000 cells per condition. (D) Rat pancreatic islets were incubated for up to 6 hours in the presence of 10 nM E2. RNA was subsequently isolated and insulin as well as cyclophilin transcript levels were evaluated by quantitative RT-PCR. Data are presented as fold change of mRNA levels as compared to control untreated islets and normalized to the cyclophilin transcript. Values represent the mean±SEM of 3 independent experiments performed in duplicates. Statistical significance was tested by Student's t test. *, P<0.05; **, P<0.01.

Intact islets of Langerhans from adult male mice were cultured in the presence of 11mM glucose and increasing concentrations of E2 for 48 hours. Insulin content increased following an inverted-U dose-dependent response (Fig. 1A) at doses similar to the physiological concentrations of E2 observed in the mouse during pregnancy [21] and in the proestrus stage [22]. Insulin content was also higher in the majority of single cultured β-cells when measured with immunocytochemistry (Fig. S1). The islet cell viability remained high and similar in both E2 and vehicle-treated islets (Fig. S2). Islet area was unchanged in E2 vs. vehicle-treated islets (Fig. 1B) and no increase on BrdU incorporation was observed, indicating no replication of β-cells in response to E2 treatment. Together, these experiments suggest that the increase in insulin content is not a consequence of an increase in β-cell mass.

We then investigated the possibility that the modulation of insulin gene expression was responsible for the increase in insulin content in pancreatic β-cells. A temporal expression profiling of the insulin mRNA was evaluated by quantitative real time PCR in cultured islets. As shown in figure 1D, insulin mRNA levels were induced 1.6 fold 6 hours after islets were exposed to 10nM E2. Semiquantitative RT-PCR experiments after 48 hours exposure to E2 gave essentially the same result (Fig. S3).

**Figure 2 pone-0002069-g002:**
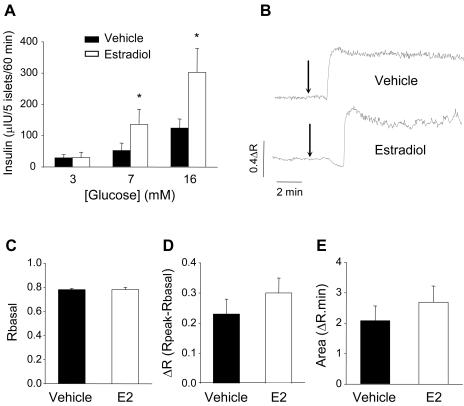
E2 action on insulin secretion and glucose-induced [Ca^2+^]_i_ signals. (A) Glucose-induced insulin secretion from islets exposed to vehicle (black column) or 1 nM E2 (white column) for 48 hours with 3, 7 and 16mM glucose. (n = 3, 5 mice), * p<0.05 Mann-Whitney test compared to vehicle. (B) Record of fluorescence vs. time from whole islets of Langerhans treated with either vehicle or E2 in the same conditions described for experiments in figures 1–3. The Ca^2+^-dependent fluorescence of Fura-2 is expressed as the ratio F340/F380 (Materials and Methods). Stimuli were applied when indicated by arrows. (C) Comparison of the fluorescence levels in the absence of stimuli between vehicle and E2-treated islets. (D) Increment of fluorescence between the fluorescence levels at the peak obtained with high glucose (Rpeak) and the basal fluorescence (Rbasal). (E) Area under the traces during 10 minutes beginning when R increases in response to glucose. At least 8 islets were used from 3 different mice. Note that no significant differences were found.

We next studied whether the aforementioned increase in insulin content by E2 is accompanied by an alteration in insulin secretion. The experiment in figure 2A shows that at various stimulatory glucose concentrations, islets incubated with 1 nM E2 for 48 hours secrete more insulin than those treated with vehicle. This increase in insulin release is not the consequence of an alteration in the stimulus-secretion coupling process, since no change in intracellular calcium concentration dynamics was observed in response to high glucose (Fig. 2B). None of the parameters analyzed showed significant differences, basal [Ca^2+^]_i_ levels in the absence of stimuli, increment of [Ca^2+^]_i_ in response to glucose (peak size) or the area under the traces, an indicator of the global Ca^2+^ amount entering into the islet (Fig. 2C–E).

These experiments suggest that physiological concentrations of E2 produce an increase in insulin gene expression and content along with an enhanced insulin release in response to stimulatory glucose concentrations. The results are consistent with the concept that increased insulin secretion in response to stimulatory glucose in E2-treated islets may be a consequence of their higher insulin content. Nonetheless, we cannot rule out the possibility that an effect of E2 on α-cells may affect insulin release [23]–[25].

### Low doses of BPA increase insulin content in whole islets of Langerhans

**Figure 3:**
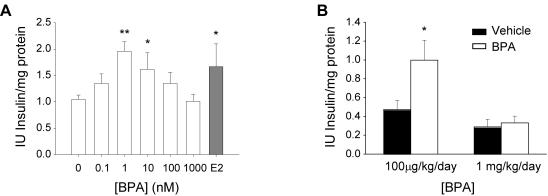
BPA action *in vitro* and *in vivo.* (A) Insulin content of islets exposed to increasing doses of BPA for 48 hours, (n = 4, 7 mice), * p<0.05. (B) Insulin content of islets obtained from two sets of experiments. First group: vehicle (black column) and BPA 100 µg/kg/day (white column) treated mice for four days. Second group: vehicle (black column) and BPA 1 mg/kg/day (white column) treated mice for four days (from at least 3 mice), * p<0.05.

To analyse if environmental estrogens act like the endogenous hormone we performed similar experiments using BPA instead of E2. Figure 3A shows a BPA-induced increase of insulin content following an inverted U-shape dose response curve, with a significant effect observed at 1 nM and 10 nM BPA when compared to vehicle exposed islets. Note that 1 nM BPA was equally effective than E2. Higher concentrations of BPA as 1 µM produced no increase in insulin content. Therefore BPA, like E2, increases pancreatic insulin content in a non-monotonic manner.

This non-monotonic behaviour of BPA occurs not only *in vitro*, but also *in vivo*. It was previously described that pancreatic insulin content increased in response to BPA *in vivo* [10]. In our study, mice were exposed daily to 100 µg/kg or 1 mg/kg BPA for 4 days. We then measured the insulin content of the islets isolated from BPA or vehicle-treated mice and found that the low dose of BPA increased insulin content while the high dose had no effect (Fig. 3B).

### Involvement of estrogen receptors in the effect of E2 and BPA

**Figure 4:**
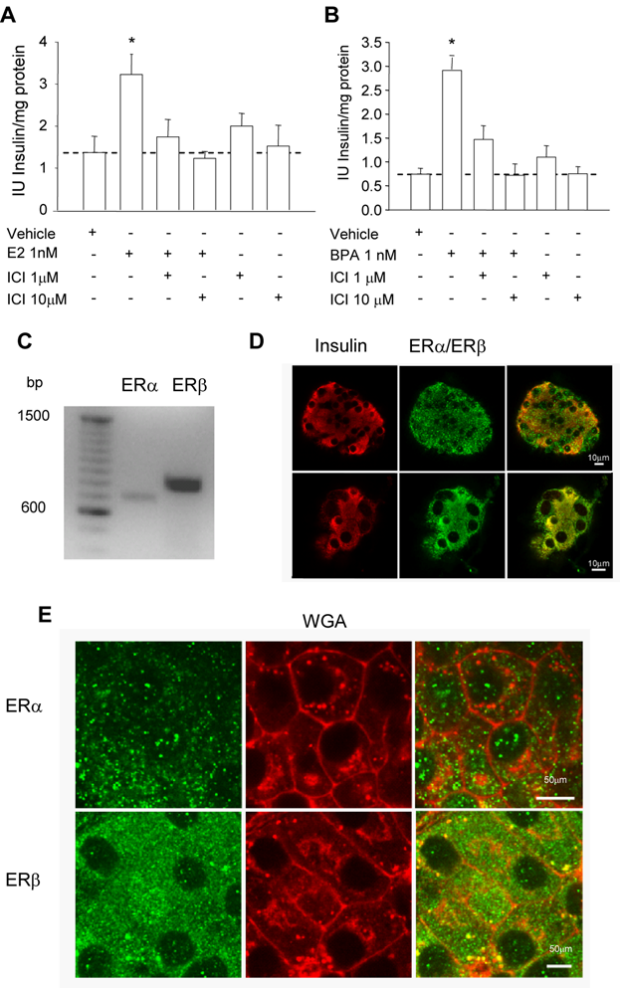
Involvement of estrogen receptors in E2 regulation of insulin content. (A) Insulin content obtained in cultured islets exposed to 1 nM E2 in the presence or absence of the antiestrogen ICI 182,780. (n≥4, from at least 4 mice), ** p<0.001, compared to vehicle. (B) Insulin content obtained in cultured islets exposed to 1 nM BPA in the presence or absence of the antiestrogen ICI 182,780. (n = 4, 8 mice), ** p<0.01, compared to vehicle. (C) RT-PCR of mRNA from fresh isolated islets was performed for both ERα and ERβ. (D) Confocal images of whole islets stained with anti-insulin (red) and anti-ERα or anti-ERβ (green). (E) ERα and ERβ subcellular localization. Single confocal images of whole islets stained with wheat germ agglutinin (red) as a marker for plasma membrane along with anti-ERα or anti-ERβ (green). Calibration bars represent 50 µm.

To determine whether the nuclear estrogen receptors mediate E2 and BPA effects in islets, we used the pure antiestrogen ICI182,780, that blocks both ERα and ERβ. Figures 4A and B show that ICI182,780 completely blocks both E2 (Fig 4A) and BPA (Fig 4B) effects in a dose dependent manner, indicating that estrogen receptors are involved.

As an initial step towards identifying the ER used by E2 and BPA to modify insulin content, the expression of ERα and ERβ was investigated in pancreatic β-cells. RT-PCR and immunocytochemistry performed on mouse islets of Langerhans confirmed that β-cells express both ERα and ERβ (Fig. 4C–D and Fig. S4). The location of estrogen receptors in subcellular organelles is important since they trigger their actions in different cellular compartments ranging from the plasma membrane to the nucleus [26]. Both receptors have been reported in the nucleus, cytosol, mitochondria and associated to the plasma membrane [27]–[32]. We studied the location of ERα and ERβ in whole islets of Langerhans using immunofluorescence and confocal microscopy. Both ERα and ERβ were present in the cytoplasm and the nucleus (Fig. 4E, Fig. S5). Overall, our data suggest that both ERα and ERβ are expressed in β-cells and localized inside and outside the nucleus.

### ERα mediates the E2 and BPA-induced increase in insulin content

**Figure 5:**
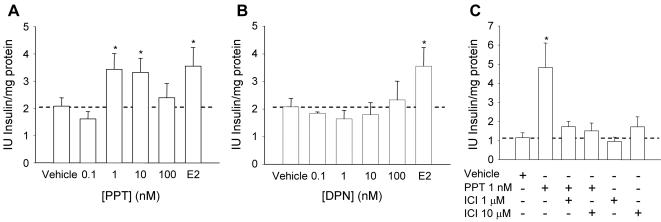
Action of ERα and ERβ agonists on insulin content. (A) Insulin content of islets cultured for 48 hours in the presence of increasing doses of the ERα-selective agonist PPT. The bar labeled E2 represents islets cultured with 1 nM E2 for 48 hours. Note that PPT increases insulin content in an inverted-U dependent manner and its potency is in the same range as E2 (n = 5, 7 mice), * p<0.05. (B) The ERβ-selective agonist DPN has no effect at doses from 0.1–100 nM. The bar labeled E2 represents islets cultured with 1 nM E2 for 48 hours (n = 4, from at least 5 mice), * p<0.05. (C) Insulin content regulation by PPT in the presence and absence of ICI182,780 (n = 4, from at least 6 mice), * p<0.05.

In order to evaluate which estrogen receptor is involved in the effect of 17β-estradiol and BPA, we used the specific agonists for estrogen receptors, PPT and DPN. The ERα agonist PPT had the same effect on insulin content as E2 (Fig. 5A). Remarkably, the increase in insulin content by PPT mimicked the inverted-U dose response described for E2. However, the ERβ agonist DPN produced no effect at any of the tested concentrations (Fig. 5B), indicating that the role of ERβ in β-cells differs from the one of ERα. The effect of the ERα agonist PPT was blocked by the antiestrogen ICI182,780 (Fig. 5C).

**Figure 6:**
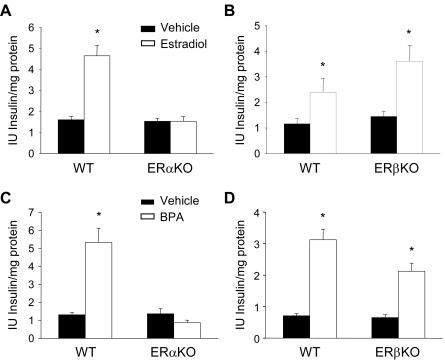
E2 effect on insulin content in ERαKO and ERβKO mice. (A) Insulin content obtained in cultured islets from wild-type and ERαKO mice exposed to either vehicle or 1 nM E2. Data obtained from 4 animals for experiments performed with wild-type mice and from 6 animals for experiments performed with KO mice, *p<0.001 compared to vehicle (B) Insulin content obtained in cultured islets from wild-type and ERβKO mice exposed to either vehicle or 1 nM E2. Data obtained from 2 animals for experiments performed with wild-type mice and 2 animals for experiments performed with KO mice, *p<0.05 compared to vehicle (C) Insulin content obtained in cultured islets from wild-type and ERαKO mice exposed to either vehicle or 1 nM BPA. Data from 4 animals for experiment performed with wild-type mice and 3 animals for experiment performed with KO mice, *p<0.001 compared to vehicle (D) Insulin content obtained in cultured islets from wild-type and ERβKO mice exposed to either vehicle or 1 nM BPA. Data from 4 animals for experiment performed with wt mice and 4 animals for experiment performed with KO mice, *p<0.001 compared to vehicle.

To unequivocally demonstrate the extent of ERα activation in regulating pancreatic insulin content we used islets from ERαKO and ERβKO mice [33]. The absence of ERα or ERβ did not affect the non-stimulated insulin content of cultured islets when compared to wild-type mice (Fig. 6A–D). The behavior in terms of [Ca^2+^]_i_ signals induced by glucose was unchanged between wild type and ERαKO/ERβKO mice (Fig. S6). This result is in agreement with the normal insulin secretion observed in islets from ERαKO and ERβKO mice [7]. Islet size was similar between islets from wild type and KO mice (12426±510 µm^2^ for ERαWT and 12123±415 µm^2^ for ERαKO; 15720±612 µm^2^ for ERβWT and 15977±764 µm^2^ for ERβKO; at least 100 islets from at least 3 different mice).

Exposure to E2 (Fig. 6A, B) or BPA (Fig. 6C, D) strongly increased insulin content in islets from wild-type mice, yet this effect was completely abolished in islets from ERαKO mice (Fig. 6A, C). This effect was observed *in vivo*, in mice treated with 100 µg/Kg/day for 4 days. No increase in insulin content was obtained in islets from E2-treated ERαKO mice (Fig. S7). In contrast, a strong increase in insulin content was observed in E2 and BPA cultured islets from ERβKO mice (Fig. 6B, D). Overall, these experiments argue strongly that ERα is the main estrogen receptor involved in the regulation of insulin content by both BPA and E2.

BPA has been considered a weak estrogen when acting through ERα and ERE. In our experiments, however, both E2 and BPA act at equal doses through ERα. It is unlikely that ERα operates via ERE, because ERE has not been described in the promoters of mouse insulin genes (types I and II) and the use of the TESS (Transcription Element Search Software) software [34] did not reveal any putative ERE. It is known that E2 works as well via alternative pathways triggered outside the nucleus that involve either PI3-Kinase or ERKs activation [35], [36].

**Figure 7:**
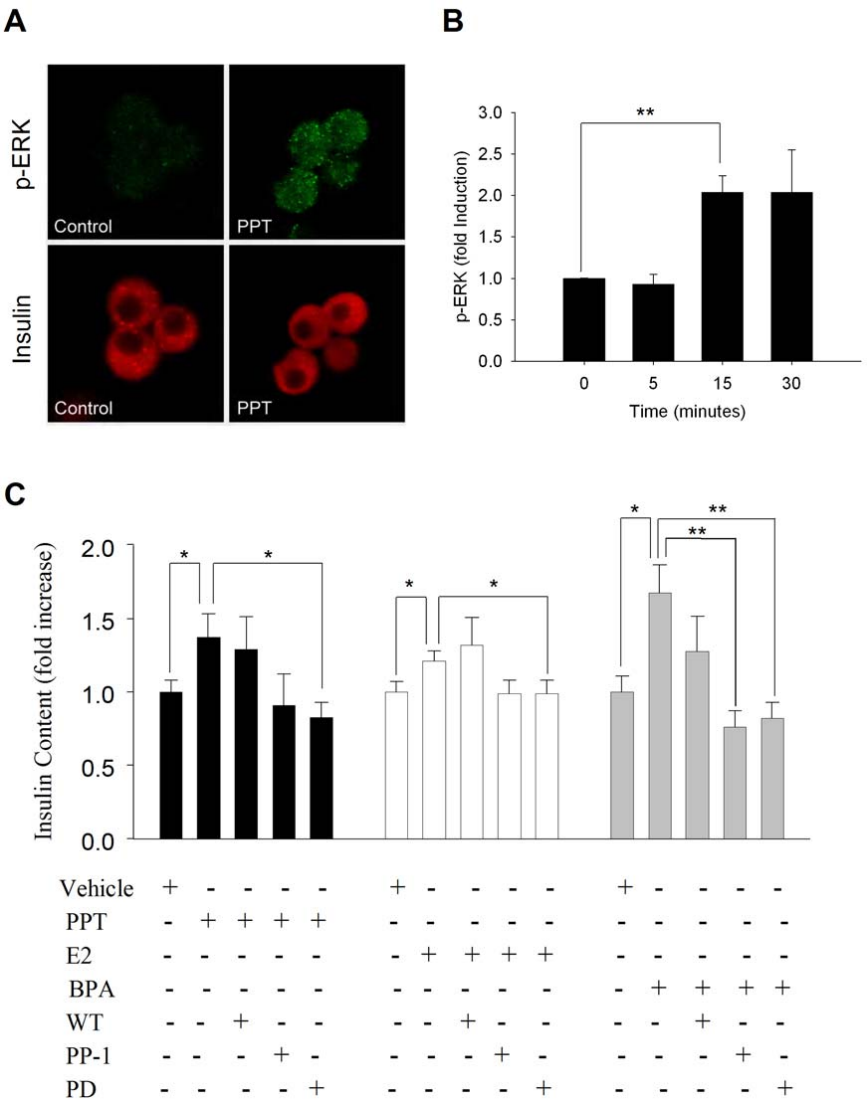
Implication of ERK1/2 in ERα action. A) Levels of ERK1/2 activation. Isolated β-cells were treated with the ERα specific agonist PPT for 0 (Control), 5, 15 and 30 min (PPT). Cells were fixed, permeabilized and incubated with antibodies to phospho-ERK1/2 (in green, p-ERK) and to insulin (in red, Insulin). Fluorescence images were registered with a confocal laser microscopy system. (B) Fluorescence intensity per cell was quantified as described in Materials and Methods. The histogram shows the fold increase in fluorescence intensity with respect to control (0 min). Data are pooled from 4 different experiments. Fluorescence intensity was obtained from at least 200 cells per condition in each experiment. *p = 0.01 Student's t-test comparing with column at 0 min. (C) Insulin content obtained in cultured islets exposed to either vehicle or 1 nM PPT (black columns), E2 (white columns) and BPA (grey columns) in the presence of the PI3Kinase inhibitor wortmannin (100 nM), the c-Src inhibitor PP-1 (10 µM) and the ERK1/2 inhibitor PD98059 (10 µM). Data were obtained from at least 4 independent experiments in duplicate using islets from at least 7 different mice. Data are expressed as fold change as compared to vehicle. *p<0.05, **p<0.01 Student's t-test comparing pairs marked by lines.

To investigate the involvement of alternative pathways, such as the MAPK one, we measured the activation of ERK1/2 in response to PPT. Using antibodies against phosphorylated ERK1/2, we obtained increased levels of activated ERK1/2 15 minutes after treatment with the ERα agonist PPT (Fig. 7A and B). Activation of ERK1/2 occurs in the cytoplasm with no apparent translocation of activated ERK1/2 to the nucleus (Fig 7A). As shown in figure 4D, ERα is located in the cytoplasm as well.

Next, we investigated the role that ERKs may have in regulating pancreatic insulin content following ERα activation. We exposed cultured islets to either 1 nM of the ERα specific agonist PPT, BPA or E2, in the presence of specific inhibitors of ERK1/2, c-Src and PI3K (Fig. 7C). The graph shows that the increase in insulin content by the three ERα ligands was completely abolished by the ERK1/2 blocker PD98059. The c-Src inhibitor PP1 showed a tendency to also inhibit the effect of the ERα agonists in insulin content, although it is only statistically significant in the case of BPA. These experiments point to the possible involvement of ERK1/2 in the ERα regulation of insulin content.

## Discussion

The present work points to a significant role of ERα in the regulation of insulin synthesis by estrogens. Long-term exposure to physiological levels of E2 and environmentally relevant doses of BPA up-regulates pancreatic insulin content through a mechanism involving activation of ERα.

Results presented here suggest that E2-induced increase of pancreatic insulin content was a consequence of an up-regulation of insulin mRNA levels rather than an increase of β-cell viability or β-cell mass.

Converging evidence from experiments performed with specific ERs agonists, PPT and DPN, the antiestrogen ICI182,780, as well as the use of ERαKO and ERβKO mice, have demonstrated that both E2 and BPA up-regulation of insulin content is mediated by ERα.

Despite the fact that ERαKO mice are insulin resistant [1], islets from both ERαKO and ERβKO mice presented normal Ca^2+^ signals in response to glucose, indicating that the stimulus secretion coupling was unaltered. Consequently, insulin secretion in response to glucose in these islets was unchanged when compared to WT [7]. Islet area was similar between WT and KO mice indicating that β-cell mass was unaffected. Furthermore, insulin content after 48 hours culture was unaltered in control conditions for both ERαKO and ERβKO mice. E2 or BPA increase insulin content in cultured islets from WT and ERβKO mice, while it remained unchanged in islets from ERαKO mice. The absence of effect of E2 in increasing insulin content in ERαKO mice was observed *in vivo* when mice were treated for 4 days.

The effect of both E2 and BPA occurs following an inverted-U shape dose-response curve, being significant at doses as low as 100pM-1 nM. The use of the specific ERα agonist PPT suggests that the sole activation of ERα is enough to provoke this inverted-U dose response. The absence of effect of high BPA exposure was observed *in vivo.* Although the action of BPA observed *in vivo* may reflect the *in vitro* effect, we must take into account that a regulation of other parameters by BPA may also contribute, these include blood glucose and insulin levels as well as insulin sensitivity. This non-monotonic response has been described in other cellular systems and has important implications for the risk assessment of endocrine disruptors, since standard testing of xenobiotic estrogenic chemicals only involves testing a few very high doses [37]–[39].

In the past, BPA was considered a “weak” estrogen because of its low binding affinity to both ERα and ERβ [18], [19], as well as for having a low transcriptional activity through these ERE binding receptors [40]. It is rather surprising that the low levels used in our work have such a large effect, the same as E2, on insulin content up-regulation. Examples of low dose effects of BPA have been reported in a wide variety of tissues and cell types [20], [37], [41], although the mechanisms of action to explain them are still unclear. One explanation to be considered is that BPA may act via ERα through mechanisms other than binding to ERE, through other transcription factors binding to their respective response elements [42]. Our data indicate that in β-cells, ERα is located inside and outside the nucleus and it is known that outside the nucleus it can activate protein kinases [43], [44]. It is therefore plausible that ERα activation by BPA occurs at low doses when the response is initiated outside the nucleus through alternative signalling pathways [26], [36], [45]. Our results indicate that ERα activation by the specific ERα agonist PPT rapidly activates ERK1/2. In addition, increment of pancreatic insulin content by PPT, E2 and BPA was blocked by the ERK1/2 inhibitor PD98059.

It is documented that insulin gene transcription is regulated by ERK1/2 [46], [47]. It is also well established that E2 induces rapid responses through alternative mechanisms that involve ERα and ERKs [26], [35], [36], [48]. Activation of ERKs by low doses of BPA has also been reported in rat cerebellar cortex [49].

In addition to the action of BPA described above, the present report demonstrates that 17β-estradiol increases pancreatic β-cell insulin mRNA levels, insulin content and insulin secretion at levels within the range reached during pregnancy and at proestrus stage [21], [22]. During pregnancy, islets adapt to the increased demand for insulin undergoing major changes in their structure and function. Among these changes, the increase of insulin synthesis [50], [51] is of great importance. Our results indicate that E2 acting through ERα may be an important hormone involved in endocrine pancreas adaptation during pregnancy.

It is important to note that E2-increased insulin content and secretion will be beneficial when it occurs during an appropriate period of time and at doses within the physiological range. This may happen during pregnancy. However, if this estrogenic action occurs at an inappropriate time, or at doses out of the physiological levels, it may cause adverse effects such as insulin resistance [10]. This may happen when there is an exposure to an environmental estrogen, such as BPA, which causes insulin resistance in healthy male mice [10]. Therefore the ERα mediated increase of insulin content by BPA in pancreatic β-cells may contribute to the BPA-provoked insulin resistance demonstrated *in vivo* [10].

## Materials and Methods

### Materials

Fura-2 AM was obtained from Molecular Probes, Inc (Leiden, The Netherlands), ICI182,780 [52], DPN (Diarylpropionitrile) [53] and PPT (propylpyrazole triol) [54], PP-1, PD98059 and Wortmannin from Tocris Cookson Ltd (Avonmouth, UK). Other substances were obtained from Sigma (Madrid, Spain).

### Animals

Swiss albino OF1 male mice (8–10 weeks old) were used. All animals were kept under standard housing conditions. A committee on internal animal care and use reviewed and approved the method used.

### ERαKO and ERβKO

Generation and genotyping of both ERβKO and ERαKO mice are described elsewhere [33]. Adult wild type, ERαKO and ERβKO animals selected for this study originated from litters of heterozygous ERα or ERβ breeding cages in a pure C57BL/6 genetic background. Institutional guidelines of the Commission d'Ethique de l'Expérimentation Animale of the University of Geneva Medical School and the Geneva Veterinarian Office were followed in the care and use of animals. Islets of Langerhans from ERαKO and ERβKO animals were treated as described below for islets from OF-1 mice.

### Islet Culture

Pancreatic islets of Langerhans were isolated by collagenase (Sigma, Madrid, Spain) digestion as previously described [55] and cultured in groups in RPMI 1640 without phenol-red, containing 11mM glucose (Cambrex,Belgium) at 37°C in a humidified atmosphere of 95% O_2_ and 5% CO_2_ for 48 h. The medium was supplemented with 10% charcoal dextran treated fetal bovine serum (Hyclone, USA), 2 mM L-glutamine, 200 U/ml penicillin and 0.2 mg/ml streptomycin. Stimuli were always present in the culture media.

### Insulin secretion and content measurement

For insulin secretion, the 48 h-cultured islets were washed during 2 hours with a buffer solution containing (mM) 120 NaCl, 5 KCl, 25 NaHCO_3_, 1.1 MgCl_2_ and 2.5 CaCl_2,_ 3 mM D-glucose; pH 7.35, when gassed with 95% O_2_ and 5% CO_2_ . Groups of 5 islets were then incubated in 1 mL of this buffer in the presence of 3, 7, and 16 mM glucose. After 1 hr, the medium was collected, and insulin was measured in duplicate samples by radioimmunoassay using a Coat-a-Count kit (DPC, Los Angeles, CA, USA).

To obtain the insulin content, the islets grouped in batches of 10 were hand picked and incubated overnight in an ethanol/HCl buffer at 4°C. At the end of the incubation period, the buffer was removed and studied for insulin content using radioimmunoassay with a Coat-a-Count kit (DPC). Protein determination was performed using the Bradford dye method. Insulin and protein content was determined in each islet sample and the ratio of both parameters was calculated for each sample. The investigator was blinded during quantification.

### Islet size

Transmission images from isolated whole islets were taken using a Zeiss axiovert 200 (Zeiss, Madrid, Spain) and a Hamamatsu Digital Camera C4742-95 (Hamamatsu Photonics, Barcelona, Spain). Mean islet area was quantified using ORCA image software from HAMAMATSU (Hamamatsu Photonics, Barcelona, Spain).

### BrdU incorporation

The islets isolated as described above were dispersed into single cells with trypsin. Cells were then centrifuged and resuspended in RPMI 1640 without phenol-red (Cambrex, Belgium) and with 10% charcoal dextran treated serum (Hyclone, USA), 2 mM L-glutamine, 200 U/ml penicillin and 0.2 mg/ml streptomycin. They were then plated on glass covers and cultured for 48h in the presence of 10 µM BrdU (Sigma, Madrid, Spain). After this, the cells were fixed 2 min with 4% PFA and washed with PBS. The cells were then treated with 2 N HCl for 20 min at 37°C and three washes with 0.1M Na_2_B_4_O_7_ (pH = 8.5). Permeabilization was achieved with 5 min treatment with 1% Triton X-100. Non-specific interactions were blocked with PBS+5% normal goat serum for 1 h. As primary antibody, mouse anti-BrdU (Dako, M0744) at 1/20 was used overnight at 4°C. As secondary Antibody, goat anti-mouse Alexa Fluor 488 was used at 1/500 for 1h at room temperature. The nuclei were stainined with 1 µM Ethidium Homidimer-1 for 15 min at room temperature. BrdU-positive cells were represented as the percentage of a total number of 1000 cells per coverslip.

### Real Time PCR

Pancreatic islets were isolated from male Wistar rats (Elevage Janvier, Le Genest-St-Isle, France) by collagenase digestion as previously described [56]. Islets were cultured for 48 hours in RPMI1640 supplemented with FBS pre-treated with charcoal. The latter eliminates endogenous estrogens remaining in FBS. Subsequently, islets were incubated in the presence of 10 nM 17β-estradiol for various times (0 to 6 hours). RNA was then extracted with the RNeasy micro kit (Qiagen,CH) and cDNA generated as previously described [56]. Primers for cyclophylin and insulin were designed using the Primer Express Software (Applera Europe, Rotkreuz, Switzerland) and sequences can be obtained on the Web page http://phym.unige.ch/groupes/gauthier/index.php. QT-RT-PCR was performed using an ABI 7500 Sequence Detection System (Applera Europe) and PCR products were quantified using the SYBR Green Core Reagent kit. Two distinct amplifications derived from at least 3 independent experiments were performed in duplicate for each transcript and mean values were normalized to the mean value of the reference mRNA cyclophilin.

### Recording [Ca^2+^]_i_

Pancreatic islets of Langerhans were isolated by collagenase digestion and cultured as described above in Materials and Methods. Islets were loaded with 5 µM Fura-2 AM for at least 1 hour at room temperature. Calcium records in the whole islet of Langerhans were obtained by imaging intracellular calcium under an inverted epifluorescence microscope (Zeiss, Axiovert 200). Images were acquired every ∼3s with an extended Hamamatsu Digital Camera C4742-95 (Hamamatsu Photonics, Barcelona, Spain) using a dual filter wheel (Sutter Instrument CO, CA, USA) equipped with 340 and 380 nm, 10nm bandpass filters (Omega optics, Madrid, Spain). Data were acquired using ORCA software from Hamamatsu (Hamamatsu Photonics, Barcelona, Spain). Fluorescence changes are expressed as the ratio of fluorescence at 340nm and 380 nm (F_340_/F_380_). Results were plotted and analyzed using commercially available software (Sigmaplot, Jandel Scientific).

### Immunocytochemistry of whole islets

Islets isolated as previously described were fixed with Bouin's solution for 5 min and washed with PBS. Then, they were dehydrated with 30, 50, and 70% ethanol, for 3 min each, and washed with PBS. After this, islets were treated with 0.5% Triton X-100 for 15 min and then washed with PBS. The non-specific staining was blocked with PBS supplemented with 0.1% Triton X-100 and 5% serum from the same host as the secondary antibody used. After the islets had been incubated for 1h at room temperature, primary antibodies were added to the blocking solution. These were: anti-insulin (1∶200, I2018, Sigma), anti-ERα, G-20 (1∶100, sc-544, Santa Cruz), and anti-ERβ, L-20 (1∶100, sc-6822, Santa Cruz). The islets were incubated overnight with the primary antibodies at 4°C, except for the anti-ERβ, which was kept for 2 days at 4°C. Secondary antibodies (Alexa Fluor® Molecular Probes) were used at 1∶1000 in PBS+0.1% Triton X-100+1% serum from the same host as the secondary antibody, for 1h at room temperature. To stain mitochondria, live islets were previously incubated with 500nM Mitotracker® Red CMXRos (Molecular Probes) for 30 min, then washed with PBS and fixed as described. To label nuclei, 1 µM ethidium homodimer-1 (Molecular Probes) was used for 15 min at room temperature at the end of the protocol. To stain the plasma membrane, 1 µg/ml wheat germ agglutinin-tetramethylrhodamine was used (Molecular Probes) and added at the same time as the primary antibody. A confocal Zeiss Pascal 5 microscope and a Zeiss 10X objective (numerical aperture = 0.3) were used too obtain images for quantification; a Zeiss 40X objective (numerical aperture = 1.3) was used for the rest. The images were analyzed using LSM Zeiss software (Zeiss, Jena, Germany). Immunofluorescence intensity was measured in the plane with maximum fluorescence. The results were expressed as average pixel intensity and normalized with respect to the mean value of the pixel intensity of vehicle-treated islets.

### In vivo experiments

BPA was dissolved in tocopherol-stripped corn oil and administered subcutaneously at the indicated concentration. The amount of vehicle was kept constant at 100 µl. Swiss albino OF1 male as well as Wild type and ERαKO male mice were injected twice a day, at 9.00 am and 8.00 pm, with 50 μg/kg or with 500 μg/kg E2 as in the experiment described in figure 3B. Islets were isolated and insulin content determined as described above.

### Viability test

The Live/Dead® Viability/Cytotoxicity kit for mammalian cells (Molecular Probes) was used to test the viability of the islets of Langerhans after being cultured for 48h. The manufacturer's instructions were followed. After 48h in culture with vehicle or 1 nM E2, islets were incubated with 2 µM calcein and 4 µM ethidium homodimer-1 for at least 30 min at room temperature, while gently being shaken. Then the islets were examined using the previously mentioned confocal microscope and the 40X objective. Images of 2.5 µm sections of whole islets were taken every 3 µm and the number of dead cells per islet were counted as nuclei stained with ethidium homodimer-1.

### Immunocytochemistry of isolated cells

#### Insulin content

The islets isolated as described above were dispersed into single cells with trypsin. Cells were then centrifuged and resuspended in RPMI 1640 without phenol-red (Cambrex,Belgium) and with 10% charcoal dextran treated serum (Hyclone, USA), 2 mM L-glutamine,200 U/ml penicillin and 0.2 mg/ml streptomycin. They were then plated on 24-well tissue culture plates and cultured for 48h. After this, the cells were washed with PBS and fixed as described previously for whole islets. The cells were first incubated with a monoclonal anti-insulin antibody (1∶200 dilution; Sigma, Madrid, Spain) for 2 hr and then for 1 hr with a secondary antibody, anti-mouse IgG-conjugated fluorescein isothiocyanate (IgG-FITC; 1∶200 dilution; Sigma), both at room temperature. The cells were washed with PBS overnight. Images were obtained with a Zeiss Pascal 5 confocal microscope using a Zeiss 20X objective (numerical aperture = 0.5) and analyzed using LSM Zeiss software (Zeiss, Jena, Germany). We measured immunofluorescence intensity in random fields. The results were expressed as average pixel intensity and normalized with respect to the mean value of the pixel intensity of vehicle-treated cells.

#### ERK1/2 phosphorylation

The islets isolated as described above were dispersed into single cells with trypsin. Cells were plated onto coverslips and incubated for 2 hours in a solution containing (mM): 115 NaCl, 5 KCl, 10 NaHCO_3_, 1.1 MgCl_2_, 10 NaHCO_3_ and 2.5 CaCl_2,_ 0.1% BSA; pH 7.35, with 11mM glucose. After this, the cells were exposed to PPT for 0, 5, 15, 30 minutes, then washed with PBS and fixed as described previously for whole islets. The cells were then permeabilized with PBS+0.5% Triton X-100 for 5min and the non-specific binding was blocked with PBS+5% normal goat serum for 1h at room temperature. The cells were first incubated with a monoclonal anti-insulin antibody (1∶200 dilution; Sigma, Madrid, Spain) and a rabbit anti-phospho-p44/42 MAPK (ERK1/2; 1∶500; Cell Signaling) for at least 48h at 4°C. Secondary antibodies (Alexa Fluor® Molecular Probes) were used at 1∶1000 for 1h at room temperature. Images were obtained with a Zeiss Pascal 5 confocal microscope using a Zeiss 63X objective (numerical aperture = 1.25) and analyzed using LSM Zeiss software (Zeiss, Jena, Germany). We measured immunofluorescence intensity in random fields. The results were expressed as average pixel intensity and normalized with respect to the mean value of the pixel intensity of vehicle-treated cells (0 min).

### RNA extraction and RT-PCR

RNA was extracted from isolated islets using a commercial kit (RNeasy, Qiagen) according to the manufacturer's instructions. Total RNA was reverse-transcribed by using the First Strand cDNA Synthesis Kit (AMV, Roche) in a total volume of 20 µl. cDNA was subjected to PCR amplification by using the Expand High Fidelity PCR System (Roche Diagnostics). 18S was used as a housekeeping gene for the semiquantitative RT-PCR. PCR primers were as follows: Insulin Forward, 5′-GAGCCCTAAGTGATCCGCTAC-3′; Insulin Reverse, 5′-CAAAGGTTTTATTCATTGCAGAGG-3′; 18S Forward, 5′-GGGAGGTAGTGACGAAAAATAAC-3′; 18S Reverse, 5′-AATCATGGCCTCAGTTCCGAAA-3′; ERα Forward, 5′-CATGGAGTCTGCCAAGGAGAC-3′; ERα Reverse, 5′-CAAGAGCAAGTTAGGAGCAAACA-3′; ERβ Forward, 5′- AGAACACACCTTGCCTGTAAAC-3′; ERβ Reverse, 5′-CCAGAATCCCTTCCACGCAC-3′. 50 ng RNA and 30 cycles were used for the insulin and 18S PCR and 250 ng and 35 cycles for the ERα and ERβ PCR. The PCR products were separated on 1–2% agarose gels and visualized by ethidium bromide staining under UV illumination.

### Statistical analysis

Data are expressed as mean±SEM. Pairwise comparisons were made using Student's t-test, unless stated otherwise. A probability level of <0.05 was considered statistically significant.

## Supporting Information

Figure S1 Increase of insulin content in isolated β-cells. Isolated β-cells were treated with vehicle or E2 for 24–48h, fixed and stained with anti-insulin antibody. Blue corresponds to low and red to high fluorescence intensity. The pixel intensity was measured and normalized. 3 mice were used and at least 150 cells/condition were counted per mouse. * p<0.01, ** p<0.001.
(8.22 MB TIF)

Figure S2 The increase in insulin content by E2 is not due to an improvement in cell survival within the islets of Langerhans. Live cells are stained with calcein (green) and dead cells are stained with ethidium homodimer-1 (red). (A) Vehicle (Control) and E2-treated islets. (B) Percentage of dead cells per islet cultured under vehicle (control) or E2 conditions. A total of 80 cells were counted per islet, 14 islets were quantified from 3 different animals.
(7.51 MB TIF)

Figure S3 E2 exposure increases insulin mRNA. (A) Isolated mouse pancreatic islets were incubated with either vehicle (black columns) or 1 nM E2 (white column) for 48 hours and then 18S and insulin mRNA levels were measured by RT-PCR. (B) Quantification analysis of 4 different experiments (6 mice), * p<0.05.
(3.97 MB TIF)

Figure S4 Presence of ERα and ERβ. Immunocytochemistry of whole islets confirmed the presence of both receptors ERα and ERβ (green). Compare with the experiment performed in the absence of primary antibodies.
(5.68 MB TIF)

Figure S5 Location of ERα and ERβ. (A) Single confocal images of whole islets stained with ethidium bromide (red) for the nucleus and anti-ERα or anti-ERβ (green). (B) Single confocal images of whole islets stained with Mitotracker® (red) for mitochondria and anti-ERα or anti-ERβantibodies (green). Calibration bars represent 50 µm
(9.35 MB TIF)

Figure S6 Glucose-induced Ca^2+^ signals in ERαKO and ERβKO islets. Islets from ERαKO and ERβKO mice show no difference in the dose-response curve to glucose compared to WT. Intracellular calcium concentration was measured in isolated islets from WT and ERαKO and ERβKO mice to different stimulatory and non-stimulatory glucose concentrations. Representative records of at least 3 islets per condition.
(8.49 MB TIF)

Figure S7 E2 treatment in ERαKO mice has no effect. Increase of insulin content in islets obtained from animals treated with 100 µg/kg/day E2 (white columns) or vehicle (black columns) for 4 days. (A) Data obtained using RIA. Data are expressed as mean±SEM from 3 independent experiments *p>0.01. (B) Data obtained using immunocytochemistry. Isolated cells were fixed and stained with anti-insulin antibody as described in Materials and Methods. The pixel intensity was measured and normalized. At least 5000 cells per each condition were counted. * p<0.00001. Islets were pooled from 3 mice.
(4.26 MB TIF)
