# Supplementary figures and images for: Pancreatic Insulin Content Regulation by the Estrogen Receptor ERα
Source: PLoS One. 2008 Apr 30;3(4):e2069. doi: 10.1371/journal.pone.0002069 (PMC2323613; doi:10.1371/journal.pone.0002069)

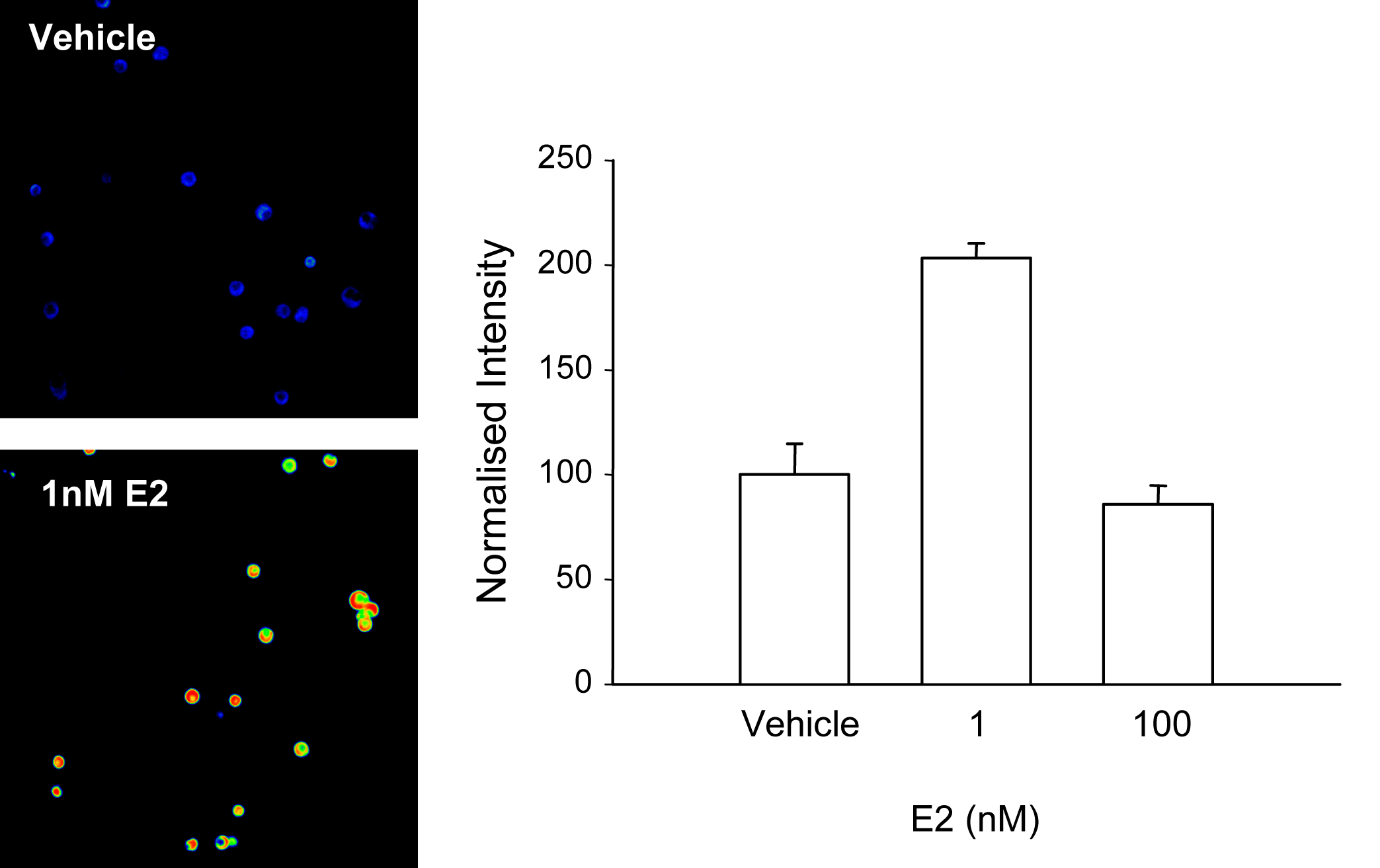

Supplement: Figure S1 — Increase of insulin content in isolated β-cells. Isolated β-cells were treated with vehicle or E2 for 24–48h, fixed and stained with anti-insulin antibody. Blue corresponds to low and red to high fluorescence intensity. The pixel intensity was measured and normalized. 3 mice were used and at least 150 cells/condition were counted per mouse. * p<0.01, ** p<0.001. (8.22 MB TIF) [file pone.0002069.s001.tif]

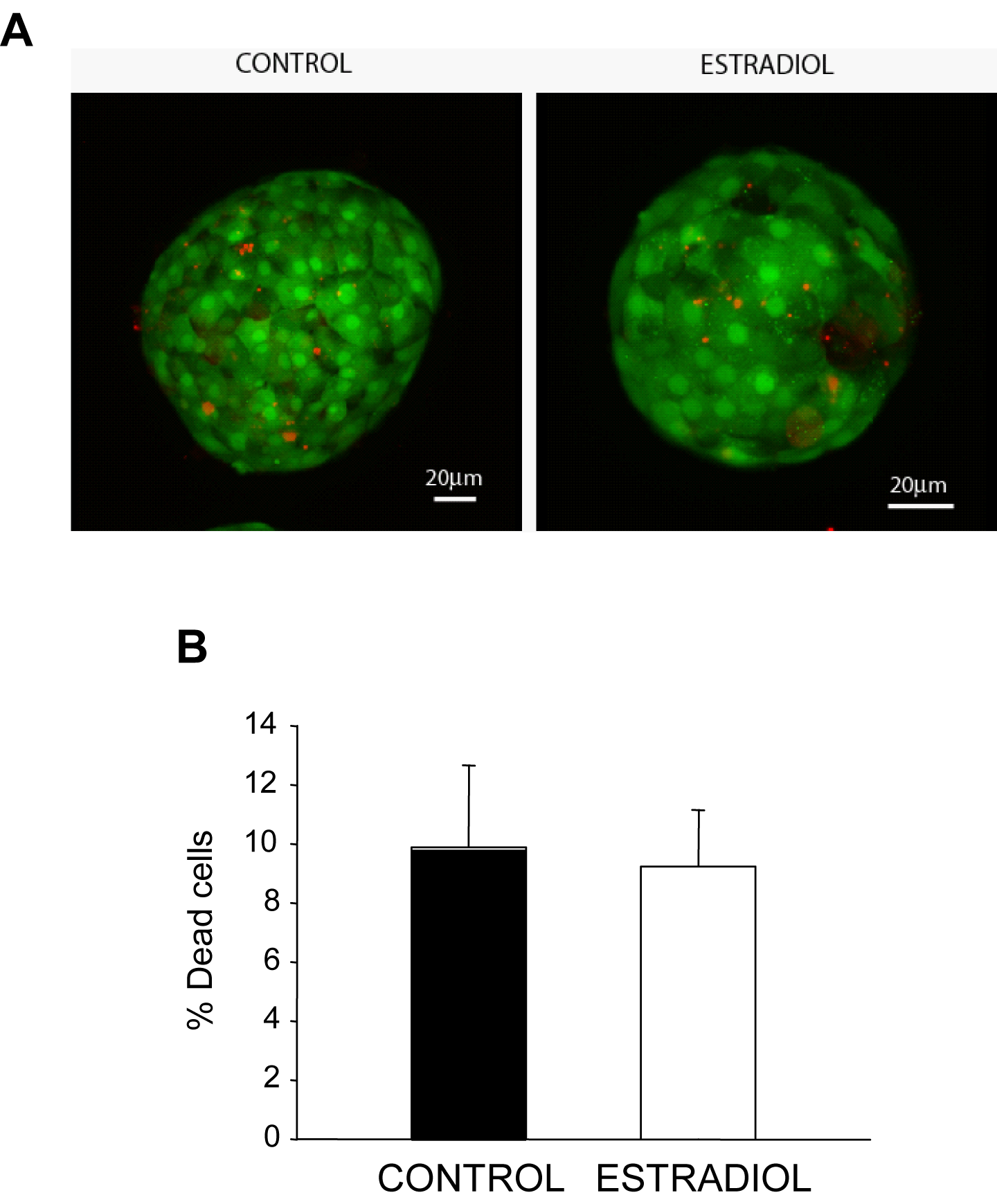

Supplement: Figure S2 — The increase in insulin content by E2 is not due to an improvement in cell survival within the islets of Langerhans. Live cells are stained with calcein (green) and dead cells are stained with ethidium homodimer-1 (red). (A) Vehicle (Control) and E2-treated islets. (B) Percentage of dead cells per islet cultured under vehicle (control) or E2 conditions. A total of 80 cells were counted per islet, 14 islets were quantified from 3 different animals. (7.51 MB TIF) [file pone.0002069.s002.tif]

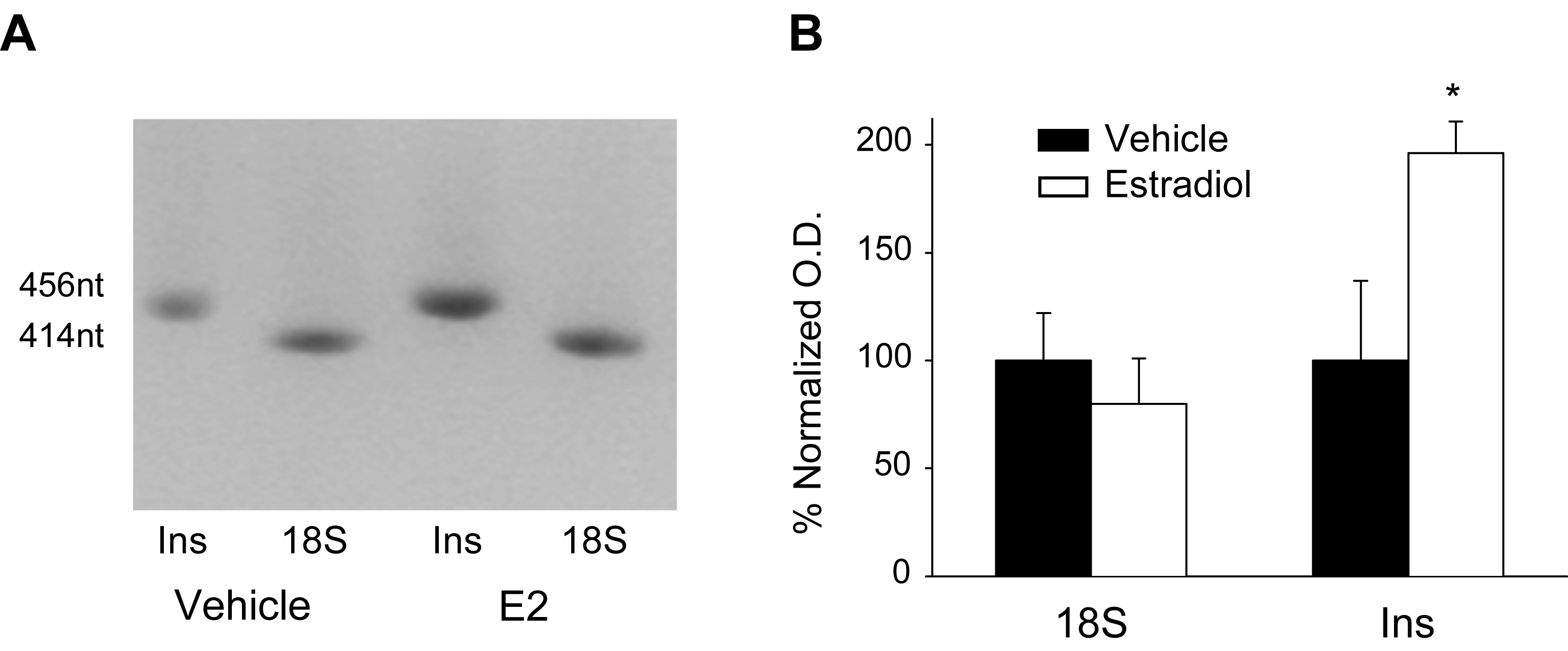

Supplement: Figure S3 — E2 exposure increases insulin mRNA. (A) Isolated mouse pancreatic islets were incubated with either vehicle (black columns) or 1 nM E2 (white column) for 48 hours and then 18S and insulin mRNA levels were measured by RT-PCR. (B) Quantification analysis of 4 different experiments (6 mice), * p<0.05. (3.97 MB TIF) [file pone.0002069.s003.tif]

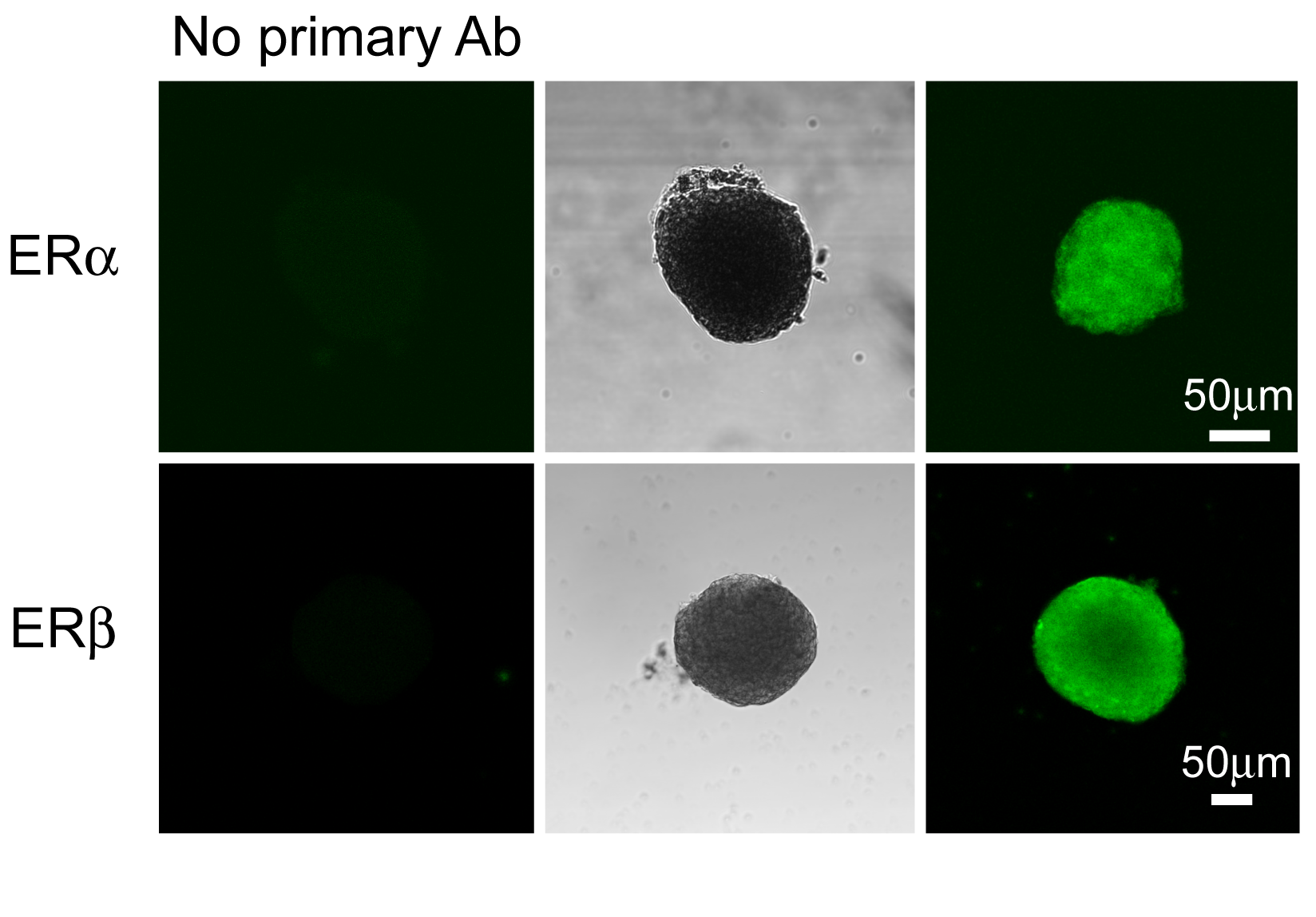

Supplement: Figure S4 — Presence of ERα and ERβ. Immunocytochemistry of whole islets confirmed the presence of both receptors ERα and ERβ (green). Compare with the experiment performed in the absence of primary antibodies. (5.68 MB TIF) [file pone.0002069.s004.tif]

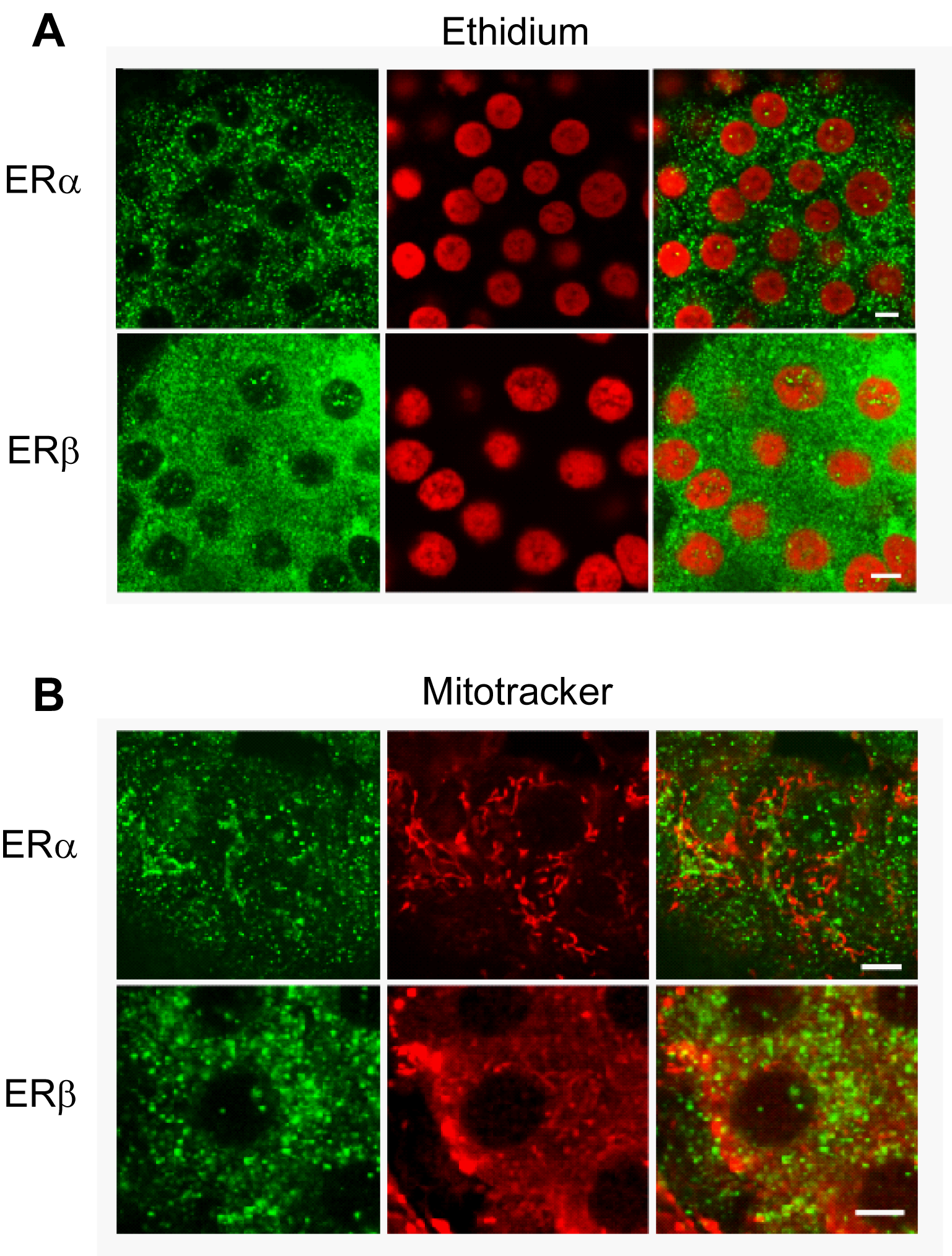

Supplement: Figure S5 — Location of ERα and ERβ. (A) Single confocal images of whole islets stained with ethidium bromide (red) for the nucleus and anti-ERα or anti-ERβ (green). (B) Single confocal images of whole islets stained with Mitotracker® (red) for mitochondria and anti-ERα or anti-ERβantibodies (green). Calibration bars represent 50 µm (9.35 MB TIF) [file pone.0002069.s005.tif]

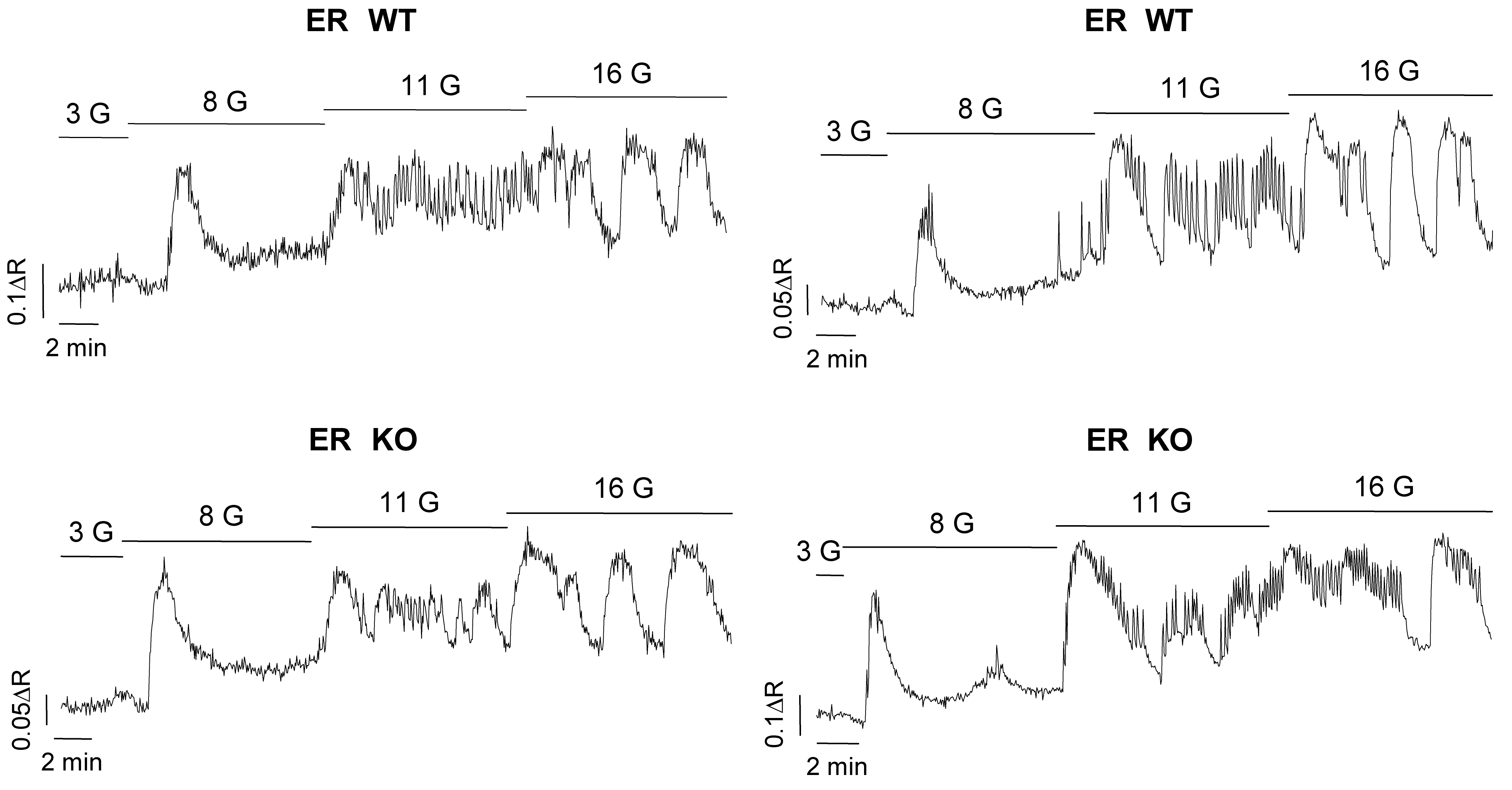

Supplement: Figure S6 — Glucose-induced Ca2+ signals in ERαKO and ERβKO islets. Islets from ERαKO and ERβKO mice show no difference in the dose-response curve to glucose compared to WT. Intracellular calcium concentration was measured in isolated islets from WT and ERαKO and ERβKO mice to different stimulatory and non-stimulatory glucose concentrations. Representative records of at least 3 islets per condition. (8.49 MB TIF) [file pone.0002069.s006.tif]

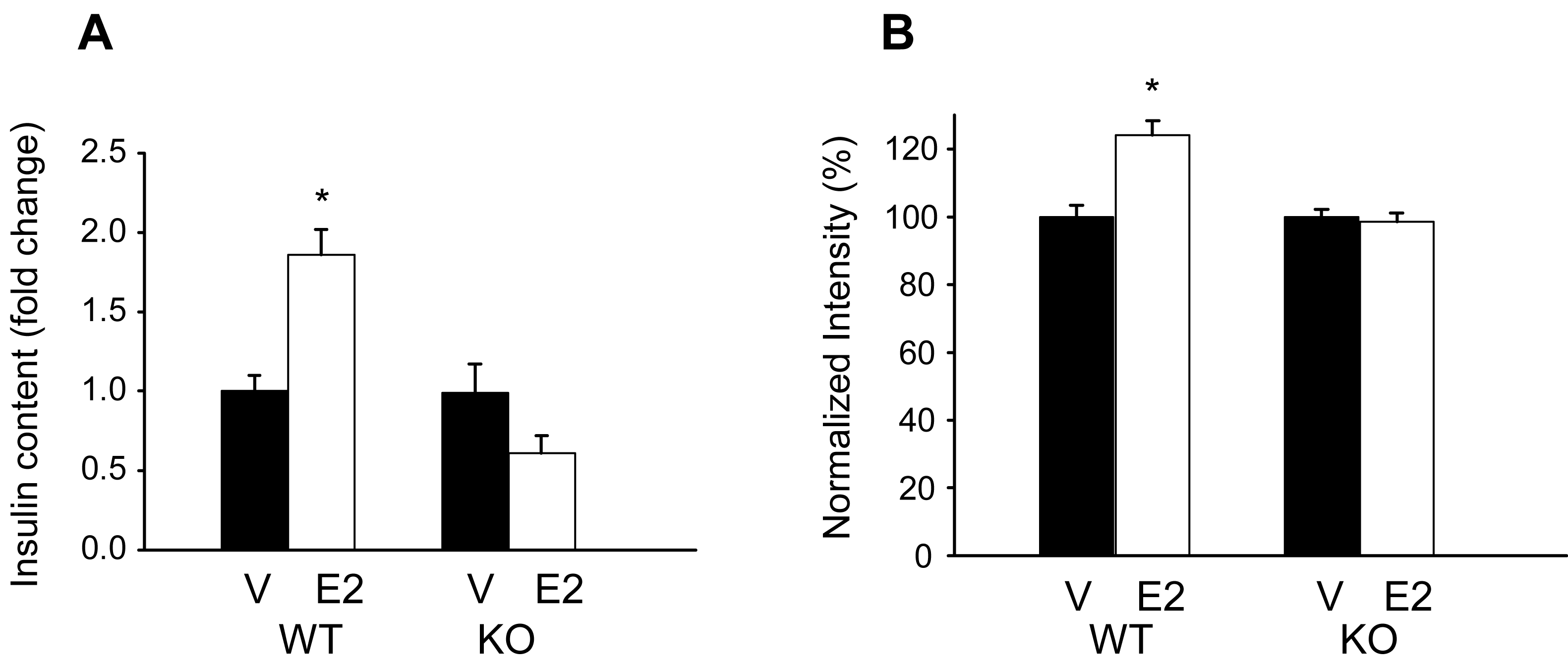

Supplement: Figure S7 — E2 treatment in ERαKO mice has no effect. Increase of insulin content in islets obtained from animals treated with 100 µg/kg/day E2 (white columns) or vehicle (black columns) for 4 days. (A) Data obtained using RIA. Data are expressed as mean±SEM from 3 independent experiments *p>0.01. (B) Data obtained using immunocytochemistry. Isolated cells were fixed and stained with anti-insulin antibody as described in Materials and Methods. The pixel intensity was measured and normalized. At least 5000 cells per each condition were counted. * p<0.00001. Islets were pooled from 3 mice. (4.26 MB TIF) [file pone.0002069.s007.tif]
